# Cancer outcome research – a European challenge Part II: Opportunities and priorities

**DOI:** 10.1002/1878-0261.13169

**Published:** 2022-01-14

**Authors:** Mette Kalager, Hans‐Olov Adami, Paul W. Dickman, Pernilla Lagergren, Karen Steindorf

**Affiliations:** ^1^ Clinical Effectiveness Research Group University of Oslo Norway; ^2^ Clinical Effectiveness research group Oslo University Hospital Norway; ^3^ Department of Medical Epidemiology and Biostatistics Karolinska Institutet Stockholm Sweden; ^4^ Surgical Care Science Department of Molecular Medicine and Surgery Karolinska Institutet Karolinska University Hospital Stockholm Sweden; ^5^ Department of Surgery and Cancer Imperial College London UK; ^6^ Division of Physical Activity Prevention and Cancer National Center for Tumor Diseases (NCT) and German Cancer Research Center (DKFZ) Heidelberg Germany

**Keywords:** cancer, prevention, quality of life, screening, treatment

## Abstract

In Part I of our review of cancer outcome research, we analysed pros and cons of various measures relevant to quantifying the burden of cancer. Based on our recommendations in Part I, we now discuss in Part II opportunities and priorities in four areas of outcome research: primary prevention; early detection screening; treatment; and quality‐of‐life assessment. We recommend the establishment of an infrastructure that facilitates high‐quality research in these areas: (a) progress in primary prevention can be assessed most directly by monitoring cancer incidence although the interpretation of temporal trends is notoriously confounded by numerous factors that complicate causal inference. (b) preventive screening, with the aim to prevent advanced disease, appears to work well in in some tumours but not in others. It will require randomized control trials (RCTs) to quantify benefits and harms although conclusive studies are increasingly difficult to undertake. We therefore propose learning screening programmes (randomization at the time of rolling out population‐based programmes) as the most feasible approach. (c) New therapeutic interventions tailored to the individual patient often require assessment in RCTs with rather complex and dynamic structure, making their design and analyses increasingly challenging but also more suited to be executed as academic, PI‐initiated trials. (d) We next discuss assessment of quality‐of‐life aspects. Quality of life is a neglected component in outcome research with an urgent need for development, validation and standardization. We finally recommend four initiatives that would pave the way for a valid and informative assessment of the goals for improved cancer control in Europe as defined by the European Academy of Cancer Sciences.

## Graphical abstract

This policy paper outlines the urgent needs and opportunities in Europe to undertake cutting‐edge cancer outcome research. Recommendations are provided for the most urgent measures in the four domains: cancer prevention, screening, treatment and quality of life. We propose an infrastructure and opportunities that can facilitate high‐quality outcome research, including learning screening programmes with dynamic randomised trials (RCTs).

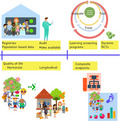

**Abbreviations**
ECIS: The European Cancer Information System
EORTC: European Organization for Research and Treatment of Cancer
FACT‐G: Functional Assessment of Cancer Therapy‐General
HPV: human papillomavirus
ICD: international classification of diseases
PSA: prostate specific antigen
RCT: randomized clinical trial
SEER: the surveillance, epidemiology, and end results
TNM: tumour, node, metastasis

## 1 Introduction

From a bird’s eyes view, improving cancer control reflects the ambition to extend, at the population level, the length of a healthy, high‐quality life with minimized suffering at an affordable cost. Such an overarching goal has, however, rarely been articulated, let alone prioritized. We are rather witnessing a focus on the fight against cancer; often, growing resources are allocated to treatments that extend life only modestly and frequently at the expense of considerable side effects and escalating costs. The Cancer Mission initiative [1] offers a unique opportunity for critical scrutiny, reconsideration of priorities and promotion of more effective use of limited resources.

The European Academy of Cancer Sciences (Academy) has established committees to provide informed opinions on specific research areas critical for the successful execution and monitoring of the progress that is expected to result from the Cancer Mission initiative.

In the present paper, the authors thoroughly review cancer outcome research, one of these critical research areas. Due to its breadth and complexity, our review has been divided into two parts. In Part I [2], we reviewed measures used to quantify the burden of cancer and to assess interventions aimed to improve cancer control. We focussed on population‐based measures of cancer burden, which can be estimated and reported for a large number of countries. These measures differ from the metrics used in clinical practice (such as caseload, case fatality rate and relapse‐free, progression‐free and overall survival), as data are rarely available at a population level to estimate these measures (relapse‐free and progression‐free survival) or are not suitable for conditions such as cancer with a long duration, where competing risks are an issue (case fatality rate).

In this Part II, we outline the research needed to achieve and assess these goals as well as the infrastructure and access to data needed to enable this research. This will permit assessment of what new insights – gained throughout the continuum of cancer research, from basic insights into the biology of cancer to implementation of effective palliative care – have contributed to the survival and quality of life of cancer patients.

Upgrading cancer outcome research in Europe offers unique opportunities to establish and validate robust cancer outcome information, given the amounting collaborative spirit and access to a population of nearly half a billion. The recommendation of the Academy to establish infrastructures of sufficient critical mass in the areas of translational research, clinical research and outcome research and promote research throughout the entire cancer research continuum is timely. As a point of departure for outcome research, substantial sources of information are already established in Europe. These resources, relevant for assessment of primary prevention, screening and therapeutic progress, are summarized in Data S1.

## 2 Outcome research for the assessment of primary cancer prevention

Obviously, preventing cancer is the first and most desired option to reduce the burden of cancer although it comes with a long lead time. No doubt, primary prevention is the ultimate success of cancer control although progress can only be shown at the population level. Declining trends in lung cancer incidence follow reduction in tobacco consumption decades later and probably represent the most convincing example. The tobacco and lung cancer experience also illustrate that it usually takes a long follow‐up time before benefits of primary prevention become apparent [3].

Reduced exposure to factors – known or unknown – causally related to malignant transformation of human stem cells (or increased exposure to preventive factors) is the fundament for primary prevention. A growing number of large, rigorously designed epidemiologic studies – with the discovery of smoking as a cause of lung cancer in 1950 as a historical landmark [4] – have led to the detection of many such factors over the last decades [5]. Several independent analyses indicate that overall cancer incidence would be reduced by up to 40% if exposure to such factors was effectively prevented [1, 6, 7, 8, 9, 10]. The European Code against Cancer established by the European Union and the WHO recommends specific actions individuals can make to reduce their risk of cancer, such as no smoking and maintaining a healthy body weight [9].

Although randomized control trials (RCTs) remain the gold standard for any assessment of cancer outcome research, such trials are rarely feasible to quantify effectiveness in primary prevention: ethical equipoise may not prevail; contamination between randomized groups and noncompliance – which is often impossible to monitor in large‐scale interventions – may entail underestimation of potential benefits; follow‐up may need to continue several decades before any benefit becomes manifest; and confounding may arise due to concomitant temporal changes in exposure to other causal factors.

Human papillomavirus (HPV) vaccination as an example of medical prevention is one of the exceptions; feasible because efficacy is plausible, and the protection provided very significant. Furthermore, timely assessment is possible since informative precursor lesions to cervical cancer are defined [11]. More than a decade did indeed pass between HPV being seriously raised as a cause of cervical cancer. In addition, it may take at least 10 years of follow‐up before benefits of vaccination become apparent with the use of surrogate endpoints; two decades are probably necessary to assess incidence and mortality, the hard outcome [11]. Similar significant reductions of liver cancer can be expected from widespread hepatitis B (HBV) vaccinations. In addition, new medical interventions, for example preventive vaccination against oncoproteins or small molecules drugs impacting on metabolism or inflammation, such as aspirin, might show effects that are substantial enough to convincingly illustrate their effectiveness in clinical trials as we have witnessed for preventive treatments of heart diseases.

Regardless of study design, cancer incidence is the most informative outcome measure to assess progress in primary prevention. Mortality, the main alternative, is conflated by prognosis, entails lower statistical power (due to fewer events) and requires longer follow‐up (Table 1). Interpretation of temporal trends in incidence is, however, often far from straight forward [2]. Such trends could be profoundly confounded – or created altogether – by changes in diagnostic intensity, introduction of more sensitive diagnostic tools, overdiagnosis attributable to screening interventions, improved cancer registration and shifts in diagnostic criteria. Hence, all these factors must be considered, adjusted for, or standardized as a prerequisite for valid comparisons between time‐periods and different countries. Outcome research is required to assess the effectiveness of preventive programmes and provide criteria for continuing or closing them.

**Table 1:** Pros and cons with different measures to assess progress in cancer control. Included are our most preferred measures although we realize several outcomes are useful. We advise the reader to read the table horizontally.

Measure	Definition	Pros and cons
Standardized mortality rate Standardized incidence rate	Number of individuals who develop the cancer or die of the cancer divided by total number at risk with each stratum (defined by age and sex) assigned weight from a defined external (hypothetical) population	*Pros:* Takes competing risk into consideration Allows unconfounded comparison with populations with a different age and/or sex distribution *Con:* Is hypothetical and will differ for any specific population depending on the standard population
Net survival	Probability of surviving beyond a given time in the hypothetical scenario where cancer is the only possible cause of death. This is the target measure of ‘cause‐specific survival’ and ‘relative survival’	*Pros:* Independent of mortality due to causes other than cancer, so is ideal for comparing survival between different populations or over time within the same population. *Cons:* Complicated definition. Hypothetical scenario is not optimal in clinical setting
EORTC QLQ‐XX	Cancer site specific modules to connect to the EORTC QLQ‐C30/ QLQ‐C15‐PAL	*Pros:* Symptoms and problems commonly occurring in the site‐specific cancer diagnosis. Modules for many different cancer diagnoses are available *Cons:* With core questionnaire together with a site‐specific module there will be 40‐60 items to reply to. Not possible to compare with other groups of people
EORTC QLQ‐C30/QLQ‐C15‐PAL	Cancer disease‐specific questionnaire with a palliative version	*Pros:* Functions and symptoms common among cancer patients in general. All cancer patients. Connected with site‐specific modules *Con:* Not possible to compare with other groups of people
In addition to the abovementioned EORTC questionnaires SF‐36/12 RAND‐36 or EQ‐5D	Generic quality‐of‐life questionnaires	*Pro:* Independent on health status. Compare different groups of people *Cos:* Lack clinically important aspects of a patients’ health EQ‐5D Commonly used in health‐economic evaluations and QALY* analyses

Finally, cost‐effectiveness has to be shown. This should include the avoided healthcare costs associated with the programmes. From a European perspective, successful outcome research requires a robust and harmonized infrastructure that covers all EU countries. This infrastructure should be composed of research units based on a uniform template but tuned to the specific needs of the various regions within EU to collect reliable data that permit monitoring of progress and comparisons between the different regions. Outcome research set‐up in this way could start monitoring and documenting the implementation and effectiveness of programmes aimed to reduce tobacco consumption, to reverse the obesity epidemic and to provide universal access to HPV vaccination throughout Europe.

## 3 Outcome research for the assessment of cancer screening interventions

### 3.1 Screening for cancer

The incontestable clinical experience that cancer patients diagnosed in early stages are more often curable than those with advanced disease has fostered attempts to broadly implement cancer screening. Although intriguing from a clinical perspective, it has proven difficult to unequivocally recognize early stages that have not yet but likely will progress to invasive and metastatic disease [10].

Firstly, the natural history of cancer from malignant transformation of one cell to clinical manifestation of cancer is usually a process spanning many years or even decades. Hence, the belief that advancement of early diagnosis through screening resulting in its detection only a few years before overt manifestation (the lead‐time) would substantially improve prognosis might be too optimistic; the malignant phenotype and its metastatic dissemination might have occurred much earlier during the natural history. Since, in a typical screened population, no more than 1 or at most 2% have the preclinical cancer of interest, the remaining majority by definition will experience no benefit. Because no screening test has 100% specificity, false‐positive findings are unavoidable, they can easily outnumber the true positive findings and they always require further diagnostic work‐up with side effects, psychological stigma, and costs. For example, there is now overwhelming evidence that both mammography screening for breast cancer [12] and PSA testing for prostate cancer [13, 14] entail substantial overdiagnosis of nonlethal disease that would not have surfaced clinically during the individual’s lifetime in the absence of screening. However, for other tumours with recurrent, and more defined precursors of tumour, such as colon and cervix cancer, screening may reduce both incidence and mortality from cancer.

Cancer‐specific mortality reduction remains the ultimate proof of benefit for early detection screening as it is not confounded by the lead time and the detection of lesions that would not have advanced (overdiagnosis) although it will be influenced by changes in cancer‐specific mortality over time in the population. It emphasizes the need for better prognostic markers that can identify early lesions that will progress to advanced disease. Testing the validity of such markers in prospective trials will be very challenging and once introduced as standard‐of‐care difficult to terminate. Validation of such markers prospectively might best be approached by learning screening programmes (see below).

### 3.2 Measuring effectiveness of new screening programmes

Randomized control trials (RCTs) potentially provide the most valid evidence of benefit. However, in the real world, such trials have been plagued by methodologic shortcomings when it comes to the assessment of new early detection tools [12]. They are often uninformative because compliance is low, participants are not screening‐naïve, control groups are contaminated or the trial design does not mimic the intended future screening programmes [15]. Ethical concerns and constrained healthcare resources rationally dictate that no screening intervention should be introduced without solid evidence of benefits outweighing harms; to initiate large‐scale human experiments without adequate scientific underpinning is simply unacceptable.

To overcome the substantial methodologic obstacles in cancer screening assessment, we propose learning screening programmes as the most realistic and valid future approach [2]. Such programmes allow timely assessment of new screening tools or strategies, for example panels of genetic markers for prostate and breast cancer screening expected to enter national screening programmes soon. A faecal DNA marker panel is already recommended by some organizations [16, 17]. Learning screening programmes are continuous testing arenas, which exploit the full benefit of knowledge generation inside the programme. When a learning screening programme has identified a new screening test, interval or threshold, it is phased in for testing. Testing involves randomized comparisons of thousands or even tens of thousands of participants with clinically relevant endpoints, such as cancer incidence or mortality. After the testing phase is over, it will be possible to assess whether the new or the old test is best. Then, the best test or method will be introduced to all. When a newer test or method becomes available for testing, the cycle begins again. Such programmes imply that randomization is used whenever new screening interventions are introduced, refined and replaced – and perhaps sometimes abandoned [14].

## 4 Outcome research for the assessment of new cancer treatments

### 4.1 Assessment of therapeutic progress

Therapeutic progress depends on unpredictable discovery and translation of basic research findings to clinical practice. Measuring therapeutic progress is not straight forward (Table 1) – except in some instances. Cure has, for example, been observed in most childhood cancers and more recently in the treatment of chronic myelogenous leukaemia with tyrosine kinase inhibitors [18] and acute promyelocytic leukaemia by retinoic acid and As_2_O_3_ [19]. In most instances, however, improved prognostic outlook is more likely to be incremental and therefore challenging to document convincingly [20, 21, 22]. Outcomes of ‘practice changing clinical trials’ have to demonstrate added value when compared to standard treatment, for patients in terms of survival and health‐related quality of life and for the healthcare organizations in terms of cost‐effectiveness. The improvement should be demonstrated on a total patient population in clinical practice as a gate‐keeper before full adoption by the healthcare system. Observational studies in large populations of patients such as long‐term follow‐up of treatment for documentation of side effects and survivorship are of critical importance.

It will also be necessary to measure the outcomes of treatment in comprehensive cancer centres (CCCs) as compared to other healthcare organizations; compelling evidence is needed to demonstrate the added value of these centres. For this, it will be particularly important but also challenging to assure that the outcomes of the patient populations that are treated in these different institutions are fully comparable. Evidently, detailed, comprehensive registries including clinical data will be required to unequivocally ascertain that patients receive the most optimal treatment resulting in the highest possible quality of life for an affordable cost. The infrastructure for outcome research has to be up to these tasks.

Even in the absence of real therapeutic progress, temporal changes in cancer patients’ survival can arise through several different mechanisms including lead‐time bias due to advancement of diagnosis (with no postponement of the date of death); overdiagnosis of non‐lethal cancer due to introduction of more sensitive diagnostic technologies; increased diagnostic activity or screening of asymptomatic individuals; and relaxed histopathologic diagnostic criteria to classify lesions as malignant ([1]; Data S1). The 6‐fold increase in the recorded incidence of malignant melanoma in the United States during the last 40 years with no concomitant trend in mortality naturally leads to an increase in survival, but without a reduction in mortality, exemplifies several of these mechanisms that make trends in cancer survival a rather unreliable measure of therapeutic progress [23].

Hence, to assess the impact of improved treatment at the population level, information on concomitant trends in incidence, mortality and survival is often necessary [2]. Monitoring trends in relevant drug use, clinical practices, treatment complications attributable to therapeutics and audits of surgical procedures can further assist the interpretation of temporal trends. To make comparisons across Europe valid and informative, the methodologic approach must be standardized and accommodate the unique features of each cancer type and site. To facilitate this, epidemiologists and biostatisticians need to work closely with clinical experts in each European country to support the establishment of standardized national clinical databases and registries.

### 4.2 The contribution of randomized control trials

Of relevance for outcome research aimed to document progress (or lack thereof), we consider compelling evidence from stringently designed and adequately powered RCTs crucial. Such trials must show that any novel therapy entails more benefit than existing alternatives and that this gain is affordable and not outweighed by side effects. Because our perspective is improvement at the population level, evidence of efficacy from RCTs is only the first step. Pragmatic RCTs are then needed to document effectiveness under real life conditions. Subsequently, improved outcomes at the population level can be achieved both by more uniform implementation of existing optimal therapies throughout Europe as well as by effective, timely translation to clinical practice of basic discoveries and novel therapies.

We emphasize that RCTs will remain fundamental for the assessment of new cancer treatments; only under exceptional circumstances will observational studies provide the evidence needed to change clinical practice. Yet, we also acknowledge that already today, cutting‐edge, rigorously designed and adequately powered RCTs with complete long‐term follow‐up are indeed challenging to undertake; only a fraction of all initiated trials manages to complete enrolment. We further predict that conducting RCTs will become even more complex in the near future. Several circumstances will likely drive this development.

Firstly, treatments with established efficacy already exist for a growing number of malignancies making placebo‐controlled trials unethical. Instead, the additional benefit of novel treatments is likely to be smaller compared with prevailing standards than with placebo. Many new RCTs will have a non‐inferiority design to test the hypothesis that a novel treatment conveys similar benefit as existing best standard but causes less harm and/or is more cost‐effective. To achieve adequate statistical power, such trials will need to be even larger than those in the past. Rapidly emerging opportunities for personalized cancer treatment – guided by molecularly defined cancer phenotypes – will further augment the need for large sample sizes allowing subgroup analyses with adequate statistical power. However, this may be offset by a greater effectiveness of treatments tailored to unique features of the tumour (e.g. with actionable driver mutations).

Secondly, long‐term follow‐up (often life‐long) of RCTs is needed to capture the entire spectrum and temporal dynamics of sequelae, side effects, quality of life (see more below), secondary malignancies, etc. Although randomization eliminates confounding at baseline, subsequent differences in treatment, lifestyle and other factors may introduce confounding between groups randomized to different primary treatments. To accommodate these complexities and allow valid statistical analyses, detailed longitudinal data collection throughout the duration of the trial will be required.

A final emerging challenge in pragmatic trials is to demystify intention‐to‐treat effects – which is prevailing standard – and improve per‐protocol analyses [24]. In brief, intention‐to‐treat analyses include all individuals randomized, independent on whether they accepted the intervention or not, comparing those randomized to the intervention arm with the control arm. The per‐protocol analysis includes only individuals who accepted the intervention, comparing individuals who accepted the intervention (compliers or attenders) in the intervention arm with the control arm. While per‐protocol analyses may introduce bias, this may also pertain to intention‐to‐treat analyses, particularly during long‐term follow‐up. Therefore, per‐protocol analyses with refined methodology may become increasingly motivated in the future because estimates are more informative and still valid. Intention‐to‐treat analyses may indeed mislead clinical practice, particularly when adherence to the assigned treatment is low; per‐protocol analyses may indeed provide more valid results in a number of instances. However, high‐quality per‐protocol analyses also require detailed collection of data throughout the follow‐up period requiring substantially more resources than those that in most instances have prevailed hitherto. Without such information, adjustment for selection bias and confounding becomes impossible [24].

## 5 Outcome research for the assessment of cancer‐related quality of life

### 5.1 Measures to improve quality of life

The need to integrate assessment of health‐related quality of life into the fight against cancer is increasingly embraced. Regulatory agencies, such as the US Food and Drug Administration and the European Medicines Agency, have begun to stress the importance of patient‐reported outcomes including symptoms and quality of life during the drug approval process [25, 26]. Such requirements have fostered the use of composite endpoints. Furthermore, both the American Society of Clinical Oncology and the European Society of Medical Oncology integrated quality of life results into their formal evaluations of clinical value of anticancer treatments [27, 28]. The importance of incorporating patients’ (or patient advocates’) preferences and needs to promote all aspects of quality of life is increasingly recognized as a fundamental component of improved cancer control and an integrated part of outcome research. In the EU Cancer Mission, quality of life and patient‐centred care will play an important role [1]. Health‐related quality of life also includes cancer survivors’ ability to resume their previous professional activities, with all its health‐economic implications. However, patient‐reported outcome measures, including quality of life, are still underused in research let alone in clinical settings.

Improved quality of life may also increase cancer‐specific survival. In clinical practice, effective cancer drugs can often not be administered as planned, for example dosages needs to be reduced or treatment paused, due to side effects. Such amendments may negatively impact treatment efficacy, while optimal management may improve patient survival, beyond clinically relevant effects on quality of life. Research on temporal dynamics or determinants of quality of life including fatigue, sleep problems and cognition is still in its infancy. We lack validated protocols to measure whether personalized treatment and management increase quality of life and lead to an optimal balance between increased survival and optimal quality of life. Further, we are short of research on consequences of cancer diagnosis among the patient’s family and friends. Their support is often crucial for the cancer patient and their own lives are usually negatively impacted by the cancer diagnosis; they might neglect themselves [29] while struggling with their daily life activities and emotional distress. Thus, the need of support for family members and friends deserves to be investigated as part of outcome research. Optimized support might also improve cancer survivorship as a whole.

We conclude that dedicated investment in quality‐of‐life research has the potential to increase survival, improve quality of life through individualized interventions during the often life‐long surveillance of cancer survivors, facilitate counselling and participation of family and friends into the process of care [30]. We do indeed believe that such a holistic approach would not only result in a more humane care of cancer patients but also ultimately save resources in our constrained health systems. Current quality‐of‐life data assessments are mostly sporadic, opportunistic, unsystematic, unstandardized, cross‐sectional without repeated measures and restricted mostly to a few cancer sites. Few objective methods are available, and no gold standards are defined.

Some instruments to measure quality of life (reviewed in Part I and summarized in Table 1) are well accepted in the community such as the European Organization for Research and Treatment of Cancer (EORTC) questionnaires and the Functional Assessment of Cancer Therapy‐General (FACT‐G) questionnaires [2]. Furthermore, what matters during treatment may differ from what is important during follow‐up. For newer treatment regimes, quality of life and side effects of cancer treatment need to be measured over a long period of time whereas more established treatment‐regimes tend to focus largely on palliative care. To guarantee sustained high quality of life among cancer survivors, we need longitudinal assessment of supportive measures including RCTs with health‐related quality of life as a primary outcome parameter. A new core questionnaire specifically for cancer survivorship currently under development by the EORTC may facilitate such research [31].

New technologies and e‐health tools, for example smartphones, tablets and wearables are now available. Patients can respond to questions directly in their daily lives, at any time and place. These technologies facilitate longitudinal monitoring and may allow even more frequent assessments. However, overly excessive and/or frequent assessments may create an undue burden to the patients and result in low compliance or reduced data quality. Therefore, timing of patient‐reported outcome assessments needs an accepted rationale. Quality of life is often measured for the first time when patients suffer from cancer symptoms and anxiety; using this as the baseline and a threshold for full recovery might be misleading. The optimal baseline assessment is in the presymptomatic phase before a cancer diagnosis and should be used as the standard for full recovery. New technologies may reduce the burden of answering questions and increase the focus on relevant information for cancer patients. Further, such technologies may facilitate assessment in the healthy population before a cancer diagnosis, representing an optimal quality of life. With computer‐ and mobile‐assisted adaptive versions, focus can be on quality‐of‐life questions of concern for a specific patient, with the option of following up with more in‐depth evaluations.

Computer‐ and mobile‐assisted flexible techniques may improve individual patient care because they facilitate management of disease‐ and treatment‐related symptoms. Data sharing between patients and healthcare professionals allows fast interactions and timely support. For example, during treatment, these technologies could facilitate assessment of quality of life and side effects regularly. If symptoms worsen or new ones appear, specific questions could be asked and action plans implemented if predefined specific threshold values are reached. European guidelines recommend continued assessment of fatigue symptoms but hardly any institution follows this recommendation [32]. Hence, new technical devices and communication technologies can greatly facilitate such monitoring and overall revolutionize our opportunities to assess quality of life and many other aspects of cancer survivorship at a much deeper level of detail than has been possible with static questionnaires.

Standardized assessments of quality of life and side effects, defined in close collaboration with patient representatives, should be incorporated into clinical routines and entered into patient charts and clinical databases. This may optimize care by facilitating follow‐up of interventions and adequate responses to side effects and reduced quality of life. However, diversity of the assessments, by, for example, instruments, timing, terminology and patient characteristics, may hinder valid comparisons between and within cancer types, treatment regimens, and within and between countries. Further research is needed on how to best structure, validate and explore databases that incorporate standard assessments but also patient‐reported outcome data with varying time intervals and depth of detail depending on the patient‐driven assessment approach.

Clear definitions and standardized terminologies are a prerequisite for valid comparisons between studies and for data mining initiatives. Also, data flow and data safety issues will become complex when patient‐generated data are combined with clinical data. Studies indicate, however, that patients are willing to share their data if data protection is secured [33]. Detailed routines and Europe‐wide population databases need to be developed to achieve the proposed public health goal to systematically evaluate the quality of life among cancer survivors after 1, 2, 5, 10 years and longer [1]. Such databases will also help to develop reference values of patient‐reported outcomes that can be used more broadly when measuring recovery in both routine care and cancer outcome research.

### 5.2 Composite endpoints

Evidence generated by cancer outcome research should guide the management of individual patients as well as the adjustment of healthcare systems. During these decision processes, we will be forced to choose between many alternatives, to navigate in the absence of solid evidence and to integrate data on three incommensurable entities namely survival, quality of life and financial expenditures. In real life, priorities are often based on the political reality of a country, the economy, the culture and individual preferences; integration of existing evidence becomes implicit rather than explicit. However, a widely acceptable composite endpoint will need to be defined to make outcome research beneficial for patients and analyses of cost‐effectiveness relevant.

Quality‐of‐life adjusted life years (QALY) gained or lost is such a measure. Some healthcare systems, for instance the National Institute for Health and Clinical Excellence (NICE) of the British National Health Service (NHS), use QALYs to determine healthcare priorities [34]. In order to generate QALY metrics, health utilities are needed; utilities are preference weights measured on a cardinal scale from zero (‘as bad as being dead’) to one (completely healthy). One quality adjusted life year is equal to 1 if health is perfect assessed with different measures (Table 1 for measures to assess quality of life). There are several issues with this measure [35, 36]. First, it is difficult to weight quality of life against survival. Second, assessment of quality of life will unlikely capture all aspects of quality of life as some might be highly individual‐specific. Third, quality of life tends to be lower among the poor, socially deprived and among disabled; as a consequence, their quality of life rating may also be lower. Fourth, relatively minor health problems may be given an unduly high weight as compared to severe problems and death, both from a patient perspective and a societal priority setting perspective [37]. Finally, the question is who should assess the ‘health’ value? The patients or the general population, cancer specialists, sociologists, professional health organizations?

Employment, depression, religion and other parameters affecting overall mortality may also be taken into account [35]. We need to establish valid and relevant composite endpoints. However, integration of all relevant measures in an optimal combined endpoint is extraordinary challenging and makes it almost impossible to define valid and relevant composite endpoints with wide applicability.

A European‐wide initiative with national representation of researchers from multiple disciplines such as the social sciences and health sector along with patients, patient representatives, clinicians and representatives from the healthy general population, could address the need for an informative and widely accepted comparable endpoint. This group could advice which core measures should be included in the combined endpoints. It will be critical to limit these to relatively universally accepted parameters in order to prevent inclusion of features that might vary strongly between geographic regions or subject to rapid change over time.

## 6 Setting priorities

### 6.1 Background

Only few European countries have nation‐wide patient registries including quality registries, and few of them provide a convenient way to access aggregated data. In 2018, 2300 oncological RCTs were initiated worldwide [38]. In Europe, the expenditure for cancer care was 103 billion Euros of which 32 billion were spent on cancer drugs [39]. This translates to 378 Euros per capita in 2018. Health expenditure has almost doubled from 1995 to 2018, whereas cancer incidence has increased by 50% from 2.6 to 3.9 million cases. More detailed cost estimates require information on date of diagnosis, diagnostics, treatment, follow‐up and longitudinally assessed quality of life. These data are crucial for outcome research that aims to document improved cancer control. They are also needed to make health‐economic analyses possible and relevant.

### 6.2 Recommendations

Promote research initiatives in outcome research to evaluate the effectiveness of primary prevention, early detection, current and future treatments, and all aspects of quality of life of cancer patients. Outcome research is indispensable to properly assess the success of the Cancer Mission and therefore should be given a high priority and adequate funding. We envision units of sufficient critical mass preferentially housed in a limited number of physical locations facilitating communication between the outcome researchers themselves as well as permitting direct interactions and exchanges with basic cancer researchers, clinicians and patients, all together with access to EU‐wide registries. There is also a need for improvements in computational infrastructure, especially network infrastructure to facilitate safe and secure data sharing but also increased investment in hardware and access to professional data managers and analysts. Suitable locations can be professional organizations focussed on (aspects of) outcome research (e.g. IARC), comprehensive cancer centres and university hospitals with a sufficiently high cancer patient population and the necessary infrastructural support. Such outcome research units will require long‐term dedicated support at a substantial level. Units operating in the different countries also need to establish close links necessary for the European‐wide alignment needed in outcome research. Initiatives to be taken include the following:

1. Establishment of population‐based cancer registration and patient registries throughout Europe. Such registration needs initially not to be nation‐wide, but it must cover a population that is sufficiently large and representative for the country to allow statistically robust analyses. The reporting should be close to inclusive; individually unique identifiers are a sine qua non for follow‐up and survival analyses.
  A team of clinicians, epidemiologists, biostatisticians and perhaps other experts should review the quality of existing life tables for the population covered by cancer registration as a prerequisite for reliable calculation of net survival in a relative survival framework. As the time for assessment of the Cancer Mission’s goals approaches, the team should also develop a detailed analysis plan that accommodates the prerequisites in each European country, while preserving comparability between countries.
2. A team of oncologists and pathologists should undertake an audit of death registration in each country, starting with contemporary standards and this should be repeated close to the time of assessment. As a priceless long‐term investment, sincere efforts should also be devoted to harmonize criteria for the classification of cancer as an underlying cause of death. Without the proposed audit, any future comparison of cancer mortality rates between countries and time‐periods will be hindered by uncertainty.
  As recommended by the Academy repeatedly, we want to emphasize the urgent need for a quantum leap towards improved, widespread, harmonized and longitudinal quality‐of‐life assessment throughout Europe. Because this task is so multidimensional – and might include development and validation of apps for smartphones and other devices – we suggest appointment of a special Task Force charged with the mission to recommend ambitious yet realistic first initiatives.
3. Initiatives to establish valid and relevant composite endpoints is a prerequisite for rational prioritization and relevant health economic analyses. The initiative should include researchers from multiple disciplines as outlined above. In addition to survival and quality of life, individuals’ preferences and values may be included.
4. Convince appropriate authorities of the importance of making population‐based data available for research while protecting individuals' rights in relation to the processing of personal data. A high‐level legal‐advice committee could play a role in identifying the legal bottlenecks and how these can be overcome.
5. Provide funding and opportunities to enrol individuals and patients in learning screening or health programmes and to include patients in academic, PI‐initiated dynamic randomized trials for new therapeutic interventions tailored to the individual patient.

## 7 Discussion

In this paper, we aimed to outline the urgent needs but also the fascinating opportunities in Europe to undertake cutting‐edge cancer outcome research. In perfect analogy to basic research with its overall rigorous process, which includes the correction of mistakes, leading to reproducible results and a deeper understanding how nature works, outcome research has to apply the same principles. Otherwise, results will not be of the quality needed to impose changes in clinical practice nor be comparable across European countries. Hence, we need convincing evidence that cancer diagnoses are correct, follow‐up complete, classification of cause of deaths standardized and population life tables relevant by showing the baseline hazard of the cancer patients in the absence of their malignancy.

Evidence generated by cancer outcome research should guide the management of individual patients as well as the adjustment of health care systems. During this decision process, we will be forced to choose between various alternatives, to navigate in the absence of solid evidence and to integrate data on survival, quality of life and financial expenditures. A widely accepted composite endpoint is urgently needed to increase the impact of outcome research and permit analyses of cost‐effectiveness. The best we can do, is to make the process explicit, transparent and receptive to critique and thereby allow continuous improvement.

RCTs will undoubtedly remain a cornerstone of cancer outcome research. Dedicated investments in infrastructure and assigning comprehensive cancer centres as coordinators and catalysts of collaboration could indeed make Europe, with access to a population of nearly half a billion, world‐leading. Timely enrolment to adequately powered, cutting‐edge RCTs in prevention, screening, treatment and quality‐of‐life research should be within reach. Realization of this vision, however, also requires recalibration of the prevailing imbalance between industry‐initiated versus investigator initiated (academic) trials to appropriately value the scholarly work of basic research discoveries, as well as translation of these discoveries to the benefit of patients, with inclusion of quality of life and cost‐effectiveness and ultimately improved public health as overarching goals.

In Europe, it is too difficult to undertake academic RCTs because of the expense and the often limited duration of guaranteed funding far too short to cover the cost associated with enrolment, follow‐up and data analyses. We also foresee further escalation of costs; non‐inferiority trials and evidence needed to show the benefits of personalized treatment will require complex trial set‐ups that may be confounded by being nonblinded, and require repeated data collection to allow proper adjustments. Furthermore, life‐long follow‐up may be needed to capture the entire spectrum of treatment sequelae and trajectories of quality of life. The bottom line of all these considerations, concerns and needs are obvious: introduction of new funding mechanisms are required to pave the road for next‐generation, innovation‐driven, cutting‐edge investigator initiated RCTs with outcome research as an integrated part. We further predict that the design of RCTs will become more sophisticated and the analyses more demanding, using adaptive trials such as basket trials, umbrella trials or platform trials and learning screening programmes are steps in the right direction. Pros and cons with a per‐protocol comparison rather than standard intention‐to‐treat analyses also deserves serious consideration [24].

## 8 Conclusions

We want to conclude our outline of opportunities and priorities with an optimistic note. Europe with about half a billion inhabitants, political freedom, growing collaboration on numerous levels and the steady increase of comprehensive cancer centres has within reach to become the global leader in cancer outcome research. Generous EU support will be of critical importance to reach this goal. This will permit strengthening of infrastructures, building of necessary capacity, and achieving the required methodologic competence in outcome research across European countries – a requirement to properly assess the progress resulting from initiatives taken in both the EU Cancer Mission and EU Cancer Plan.

## Conflict of interest

The authors declare no conflict of interest.

## Author contributions

All authors contributed to all phases in the preparation of this manuscript including design of its structure, writing of its contents and several rounds of editing before it was unanimously approved.

## Supporting information


**Data S1**. The demanding prerequisites for complete, population‐based, high‐quality cancer registration were briefly outlined in Part I [2].
