# Supplementary material for: Cancer outcome research – a European challenge Part II: Opportunities and priorities
Source: Mol Oncol. 2022 Jan 14;16(12):2300–11. doi: 10.1002/1878-0261.13169 (PMC9208079; doi:10.1002/1878-0261.13169)
Supplement: Supplementary file 1 — Data S1. The demanding prerequisites for complete, population‐based, high‐quality cancer registration were briefly outlined in Part I [2]. [file MOL2-16-2300-s001.docx]

1. **SUPPLEMENTARY MATERIAL**

**Incidence**

The demanding prerequisites for complete, population-based, high-quality cancer registration were briefly outlined in Part I [2]. Registration of the number of newly diagnosed cancers by sex, age and year in a defined population is fundamental for proper quantification and monitoring of the cancer burden, setting priorities for primary prevention, rational planning and allocation of resources for diagnosis, treatment and follow-up – and ultimately for improved cancer control. Preferably, each country should have at least one registry using world-wide accepted standards to register personal identifiers, date of cancer diagnosis, site of cancer, pathology reports including morphology codes (ICD-O-3). Recording of this basic information dramatically improves opportunities for cancer outcomes research. But reliable death registration and life tables for the entire population covered by cancer registration is also needed for survival analyses.

Additional information on clinical and/or pathological staging is not a high priority to assess whether the Academy’s goals have been achieved. Collecting such information based on standardized criteria that prevent stage shift over time due to use of more sensitive diagnostic procedures is demanding [40]. With this caveat, information on stage may substantially facilitate interpretation of trends. For example, increasing overall incidence with no concomitant trend in the occurrence of advanced/metastatic disease and no evidence of therapeutic progress, suggest that upward temporal trends in incidence are spurious and attributable to increased diagnostic activity with overdiagnosis of non-lethal cancers [41].

Whilst the TNM-system [42] is most widely used, some cancer registries, such as SEER in the US, the registries of Norway and Finland, have their own staging system (localized, regional, distant, and unknown tumor stage). This simpler system may have benefits compared to the TNM staging, because it is more stable over time – i.e. less influenced by novel diagnostic technologies - and might be more valid when historical and more recent stage specific cancer incidence and survival data are compared.

The European Cancer Information System (ECIS) provides cancer incidence, mortality and survival statistics from 27 European countries. Not all cancer sites are included in this registry: female breast, cervix uteri, corpus uteri, ovary, penis, and prostate are not covered for any of the outcomes. Several other cancers are not covered for some of the outcomes. Data can be accessed online at the ECIS homepage [43]. The outcomes available are:

1. Historical data on incidence and mortality, where the time period varies in different countries/registries. Information includes country or registry, sex (male, female, both), cancer site, 5-year age-groups, and time period.
2. Estimated incidence and mortality for year 2020. All estimates are based on historical incidence data which varies between countries/registries; some on historical incidence data and mortality data, some on mortality data only, whilst some are based on extrapolation of other countries incidence data [44].
3. Survival information for patients diagnosed 2000 to 2007, including 1-, 2-, 3-, 4- and 5 year relative-survival, age-specific and age standardized observed and relative survival, age groups (15-44, 45-54, 55-64, 65-74, 75+), sex (male, female).

National cancer registries exist in the Nordic and Baltic countries, Austria, Slovenia, Slovakia, Czech Republic, Croatia, Ireland, and Malta. Some countries use federal (Sweden, Netherlands, Germany, England and Wales), and local registration (for example France, Italy, Switzerland and Spain) [45]). *Cancer Incidence in Five Continents,* a collaboration between the International Agency for Research on Cancer (IARC) and the International Association of Cancer Registries, estimates the cancer burden worldwide. A total of 149 population-based cancer registries including 34 European countries have reported data [46]. The last volume (volume XI) covers cancers diagnosed from 2008 to 2012. Online analyses are available by cancer site and type (ICD-10), population (continent, country), age (5-year age group), and sex [46].

Information on childhood cancers is available from International Incidence of Childhood Cancer 3, based on different registries in different time periods [47]. The European Cancer Registry-based Study on Survival and Care of Cancer Patients, Eurocare, dataset contains data from 186 rare cancers (hematological malignancies, central nervous system, digestive, genital, head and neck, endocrine, thoracic, mesothelioma, embryonal tumors, sarcomas and neuroendocrine tumors). Data on incidence, prevalence and survival are available at the homepage of Rarecancernet [48].

**Mortality**

In addition to cancer registries, cause of death registries are essential and indeed needed for survival analyses. In the absence of cancer registration, cancer mortality provides the best alternative measure of the cancer burden although the specific contributions of incidence and prognosis cannot be separated. Since 2011 reporting of deaths, underlying causes, age and country is mandatory for all EU member states [49]. In 2015, the completeness of death registration including cause of death information was 99.93% [50]. However, correct classification of cause of death is challenging; both under- and overreporting of cancer as underlying (primary) cause will occur [2]. In Europe, online access to data on causes of death coded according to the ICD-10 classification system can be found in the *Eurostat database* [51]. In addition, several countries provide access to online cause of death registries as well as population registries.

**Survival**

As discussed in detail in Part I, three widely used survival measures are recommended because they provide complementary information (Table 1).

1. *All-cause survival* defined as the probability of surviving beyond a given time without dying of any cause.
2. *Net survival* – our recommended measure to assess the Academy’s goal - defined as probability of surviving beyond a given time without dying of the specific cancer of interest in the hypothetical scenario where the cancer of interest is the only possible cause of death because competing risks are ignored.
3. *Crude survival* defined as the probability of surviving beyond a given time without dying of the specific cancer of interest in the real-world scenario where it is possible to die from other causes of death.

All these measures require data on cancer incidence and date of death. Calculation of net and crude survival requires additional information on either cause of death (if using cause-specific survival framework) or valid estimates of the expected survival in the absence of cancer (if using relative survival framework) [2].

In Europe, relative survival for patients diagnosed 2000 to 2007 is available at the ECIS homepage [43], and the European Cancer Registry-based Study on Survival and Care of Cancer Patients, Eurocare, provides trends in survival and population life tables from 1983 to 1994 (Eurocare-3) [52], from 1995 to 1999 (Eurocare-4) [53], and from 2000 to 2007 (Eurocare-5) [54]. Eurocare-5 include data from more than 21 million patients with a recorded cancer diagnosis in 30 European countries. In addition, the Association of Nordic Cancer Registries has published data on incidence, mortality and survival for Denmark, Finland, Iceland, Norway, Sweden, Faroe Islands and Greenland [55]. Country specific life table data from 2012-2016, by age (0-1 year, thereafter 5-year age groups), sex and year is available at the World Health Organization home page [56]. Population life tables for the 322 population-based registries in 71 countries included in the CONCORD-3 study [57] are available at <https://csg.lshtm.ac.uk/life-tables/>

**Limitations of available data**

The EU project European Cancer Information System (ECIS) which provides information on cancer incidence, mortality and survival for 27 European countries as well as the *Eurostat database* [51] are steps in the right direction with data accessible online. However, incidence, mortality and survival statistics are not continuously updated, there is lack of granularity of age and socioeconomic status and most European countries lack centralized nation-wide cancer registries. Reporting of incident cancers is often incomplete, whilst central facilities for computerization, quality checks, regular processing and publishing of cancer incidence data and secure long-term storage remains to be established.
